# Faldaprevir for the Treatment of Hepatitis C

**DOI:** 10.3390/ijms16034985

**Published:** 2015-03-04

**Authors:** Tatsuo Kanda, Osamu Yokosuka, Masao Omata

**Affiliations:** 1 Department of Gastroenterology and Nephrology, Chiba University, Graduate School of Medicine, 1-8-1 Inohana, Chuo-ku, Chiba 260-8670, Japan; E-Mail: yokosukao@faculty.chiba-u.jp; 2 Yamanashi Hospitals (Central and Kita) Organization, 1-1-1 Fujimi, Kofu-shi, Yamanashi 400-8506, Japan; E-Mail: momata-tky@umin.ac.jp; 3 University of Tokyo, 7-3-1 Hongo, Bunkyo-ku, Tokyo 113-8655, Japan

**Keywords:** chronic hepatitis C, direct-acting antivirals, faldaprevir, genotype 1, protease inhibitor

## Abstract

The current treatments for chronic hepatitis C virus (HCV) genotype 1 infection are combinations of direct-acting antivirals, and faldaprevir is one of the new generation of HCV NS3/4A protease inhibitors. At the end of 2013, the US Food and Drug Administration (FDA) approved the HCV NS3/4A protease inhibitor simeprevir and the HCV NS5B polymerase inhibitor sofosbuvir. Simeprevir or sofosbuvir in combination with pegylated interferon and ribavirin are available for clinical use. Faldaprevir, another HCV NS3/4A protease inhibitor that also has fewer adverse events than telaprevir or boceprevir, is under development. Of interest, faldaprevir in combination with pegylated interferon and ribavirin, and interferon-free treatment with faldaprevir in combination with deleobuvir plus ribavirin provides high sustained virological response rates for HCV genotype 1 infection. The aim of this article is to review these data concerning faldaprevir. Faldaprevir in combination with pegylated interferon and ribavirin treatment appears to be associated with fewer adverse events than telaprevir or boceprevir in combination with pegylated interferon and ribavirin, and may be one of the therapeutic options for treatment-naive patients with HCV genotype 1. The interferon-free combination of faldaprevir and deleobuvir with ribavirin was effective for HCV genotype 1 infection and may hold promise for interferon-ineligible and interferon-intolerant patients.

## 1. Introduction

Hepatitis C virus (HCV) infection is a major public health problem, causing chronic hepatitis, cirrhosis and hepatocellular carcinoma (HCC), with an estimated 180 million infected people worldwide [1]. HCV is a leading cause of death from liver disease and of liver transplantation in the US [2]. The current treatment for HCV genotype 1 infection is combinations of direct-acting antivirals and faldaprevir is one of the new generation of HCV NS3/4A protease inhibitors.

The combination of pegylated interferon and ribavirin for 48 weeks led to only ~50% sustained virological response (SVR) in patients with HCV genotype 1 [3]. In 2011, telaprevir and boceprevir were licensed in use for HCV genotype 1-infected patients. The previous treatment for HCV genotype 1 infection consisted of the addition of direct-acting antivirals (DAAs) such as telaprevir or boceprevir to the pegylated interferon and ribavirin regimen [4]. These treatments could lead to higher SVR in HCV genotype 1-infected patients [4]. SVR rates among treatment-naïve patients or previous-treatment relapsers, respectively, were ~70% or 70%−88% in telaprevir-including regimens; however, those rates in patients with no previous response were ~30% [5,6,7,8,9,10,11]. Using boceprevir-including regimens, the SVR rates among previous-treatment relapsers or patients with prior nonresponse were 69%–75% or 40%–52%, respectively [5,12,13]. Telaprevir and boceprevir, first-generation DAAs, are HCV NS3/4A protease inhibitors. At the end of 2013, the US Food and Drug Administration (FDA) approved the HCV NS3/4A protease inhibitor simeprevir and the HCV NS5B polymerase inhibitor sofosbuvir. Simeprevir or sofosbuvir in the combination with pegylated interferon and ribavirin are used in daily clinical practice [14,15]. Faldaprevir is a second-generation HCV NS3/4A protease inhibitor with significant improvement in potency and adverse event profile compared with first-generation protease inhibitors. In the near future, other next-generation HCV NS3/4A protease inhibitors such as faldaprevir (BI 201335) and vaniprevir, might become available for use in association with pegylated interferon and ribavirin [16].

## 2. HCV Genome and Its Coding Proteins

HCV is a single-stranded positive RNA virus belonging to the genus Hepacivirus, a member of the Flaviviridae family ~9600 nt in size. The HCV genome contains a 5'untranslated region (5'UTR), a single open reading frame, and a 3'UTR. A single polyprotein is translated from its genome and is processed by HCV-encoding proteases such as HCV NS2 cysteine protease and HCV NS3/4A serine protease as well as host transpeptidases, into four structural (core, E1, E2 and p7) and six nonstructural (NS2, NS3, NS4A, NS4B, NS5A and NS5B) proteins [3]. However, opinions may differ regarding p7, as the function of p7 is uncertain [17,18]. The HCV RNA replication complex develops in the endoplasmic reticulum, where HCV replication occurs. Then, HCV virions are produced and released from hepatocytes into the blood. Candidate cell surface receptors for HCV are CD81, low-density lipoprotein receptor (LDLR), scavenger receptor class B type I (SR-BI), occludin (OCLN) and claudin-1 (CLDN1) [19]. DAAs against HCV or host-targeting agents (HTAs) target one of the HCV proteins or specific host proteins, respectively. These drugs strongly inhibit HCV replication [20].

## 3. HCV NS3/4A Protease Inhibitors

HCV NS3/4A protease inhibitors interfere with HCV replication by binding to HCV NS3/4A protease. These protease inhibitors may be divided into two classes depending on the nature of the active site binding group [21]. Telaprevir or boceprevir contains an α-ketoamide, which forms a covalent reversible interaction with the active site serine of the HCV NS3/4A protease catalytic triad. The other class of HCV NS3/4A protease inhibitors, including faldaprevir, contains functional groups forming ionic interactions with residues of the catalytic triad and thus exclusively non-covalent interactions with the HCV NS3/4A protease [21].

The HCV NS3/4A protease inhibitor BILN 2061 was the first agent *in vivo* to specifically target HCV replication, thus demonstrating the proof-of-concept for the inhibition of HCV NS3/4A protease as a method for suppressing HCV replication [22]. However, cardio-toxicity hampered the development of BILN 2061 [23]. Although telaprevir and boceprevir are used against HCV genotype 1 in combination with pegylated interferon and ribavirin in clinical daily practice, these first-generation HCV NS3/4A protease inhibitors are accompanied by significant adverse events, such as skin rash, anemia and gastrointestinal symptoms [24]. Thus, next-generation HCV NS3/4A protease inhibitors with fewer adverse events and improved efficacies are needed.

## 4. Faldaprevir

Faldaprevir is a potent HCV NS3/4A protease inhibitor that has completed phase 3 clinical trials in combination with pegylated interferon and ribavirin [25,26,27], as well as phase 2 assessment with the HCV NS5B polymerase inhibitor deleobuvir (BI 207127) with or without ribavirin in interferon-free regimens [28,29]. 

**Figure 1:**
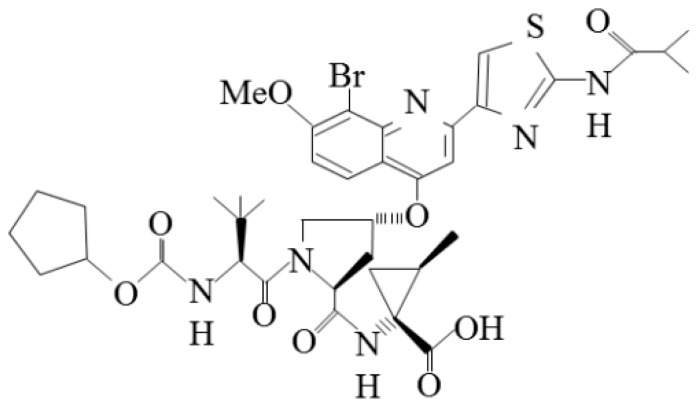
Chemical structure of faldaprevir.

The structure of faldaprevir is shown in Figure 1. Faldaprevir is a peptidomimetic HCV-specific protease inhibitor with high *in vitro* activity against HCV subgenotypes 1a and 1b, with EC50 values of 6.5 and 3.1 nM, respectively [24]. The results from a phase 1b trial [24] showed that 48–240 mg faldaprevir QD induced a rapid, dose-dependent decrease in plasma HCV RNA by >2 log10 from baseline in all patients when given QD as monotherapy in treatment-naïve patients for 14 days [24]. Sequence analysis of viral isolates from one patient obtained at baseline revealed a variant encoding an HCV NS3 V/I170T substitution that conferred a seven-fold reduction in faldaprevir sensitivity (increased EC50) relative to the subtype reference, and this patient, who was treated with 20 mg faldaprevir, had failed to achieve >2 log10 viral load reduction within the first 14 days [24]. In virological breakthrough-patients treated with triple therapy with faldaprevir, pegylated interferon and ribavirin, HCV NS3 R155K and D168V/E were the most frequently observed resistant variants in HCV subgenotypes 1a and 1b, respectively [24]. R155K variants conferred reductions in sensitivity to faldaprevir with EC50 values of 1.8–6.5 μM, whereas the EC50 values for D168V variants were 3.6–15 μM [24]. These variants have been observed with other HCV NS3/4A protease inhibitors and should confer cross-resistance to other HCV NS3/4A protease inhibitors [16]. It was reported that, in contrast to macrocyclic and covalent HCV NS3/4A protease inhibitors, changes at V36, T54, F43 and Q80 did not confer resistance to faldaprevir [30].

At the 240 mg once-daily dose, faldaprevir is a weak inhibitor of p450 (CYP)2C9, and a moderate inhibitor of CYP3A4 [31]. Sabo *et al*. [31] reported that the faldaprevir 120 mg once-daily dose has a relatively low potential for drug-drug interaction with CYP isoforms in HCV-infected patients, who frequently require medications to treat concomitant diseases or condition [32,33,34]. These interactions result in fewer adverse events and less frequent discontinuation of treatment using faldaprevir plus pegylated interferon/ribavirin [35].

### 4.1. Faldaprevir with Pegylated Interferon and Ribavirin for HCV Genotype 1-Infected Patients

For HCV genotype 1-infected patients, the following two methods have been developed: (1) triple therapy with faldaprevir, pegylated interferon and ribavirin for a total 24 of weeks [25,26,27]; and (2) interferon-free regimens with faldaprevir and deleobuvir with or without ribavirin [28,29].

**Figure 2:**
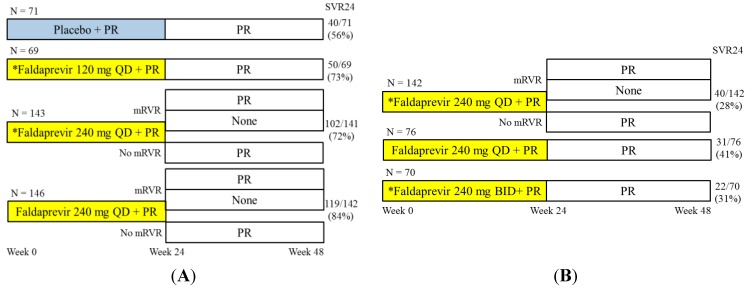
Safety, and antIviraL Effect of faldaprevir iN hepatitis C (SILEN) trial designs and results. (**A**) The SILEN-C1 trial consisted of faldaprevir combined with pegylated interferon alfa-2a and ribavirin in treatment-naive patients with chronic genotype-1 hepatitis C virus (HCV) [25]. A total of 429 treatment-naive patients without cirrhosis were randomized 1:1:2:2 to receive 24 weeks of pegylated interferon alfa-2a and ribavirin (PR) in combination with placebo (*N* = 71), faldaprevir 120 mg once daily (QD) with 3 days of PR lead-in (LI*) (*N* = 69), 240 mg QD with LI (*N* = 143), or 240 mg QD without LI (*N* = 146), followed by an additional 24 weeks of PR. The rates of sustained virological response 24 weeks after therapy (SVR24) are indicated. mRVR, maintained rapid virological response defined as HCV viral load (VL) below the lower limit of quantification (LLOQ) at week 4 (HCV RNA < 25 IU/mL) and undetectable from week 8 to week 20 (HCV RNA < 17 IU/mL). Randomization 1:1 of patients with mRVR to 24 weeks *versus* 48 weeks of PR; (**B**) The SILEN-C2 trial consisted of faldaprevir combined with pegylated interferon alfa-2a and ribavirin in chronic HCVgenotype 1-infected patients with prior nonresponse [26]. A total of 290 noncirrhotic patients with prior null (<1 log_10_ VL drop at any time during treatment) or partial response (≥1 log_10_ VL drop but never undetectable during treatment) were randomized 2:1:1 to receive 48 weeks of PR in combination with faldaprevir 240 mg QD with 3 days PR lead-in (LI) (*N* = 142), 240 mg QD without LI (*N* = 76), or 240 mg twice daily (BID) with LI (*N* = 70).

Preclinical and human pharmacokinetic (PK) studies showed that faldaprevir has a long half-life, consistent with QD dosing [24,25]. In the Safety, and antIviraL Effect of faldaprevir iN hepatitis C (SILEN-C1) trial [25], a phase 2b, multicenter, randomized, double-blind study of faldaprevir or placebo in combination with pegylated interferon and ribavirin in treatment-naïve HCV genotype 1-infected patients, 429 treatment-naive patients without cirrhosis were randomly assigned into one of four groups (Figure 2A). The rates of sustained virological response 24 weeks after therapy (SVR24) of each group are also shown in Figure 2A. SVR rates were 56% and 72%–84% in the placebo and faldaprevir groups, respectively. Ninety-two percent of patients treated with faldaprevir 240 mg QD and maintaining rapid virological response (mRVR) defined as an HCV viral load (VL) below the lower limit of quantification (LLOQ) at week 4 (HCV RNA < 25 IU/mL) and undetectable from week 8 to week 20 (HCV RNA < 17 IU/mL) achieved SVR, irrespective of the treatment duration of pegylated interferon and ribavirin. In the faldaprevir 240 mg QD without lead-in group [25], SVR rates were 82% and 84% in HCV subgenotype 1a and 1b individuals, respectively, and SVR rates were 100% and 71% in the IL28B CC and the CT/TT groups, respectively. The rates of virological breakthrough during faldaprevir treatment were 3%–6% and were predominantly associated with the selection of the HCV NS3 R155K or D168V variants for HCV subgenotype 1a or 1b patients, respectively. R155K and D168V were selected in HCV subgenotype 1a (8 of 12) or 1b relapsers (11 of 19), respectively. There were two GT1a patients with R155K and four GT1b patients with D168V resistance substitutions detected at baseline, before treatment in the faldaprevir treatment groups, and five of these six patients achieved SVR [25]. More sensitive ultra-deep sequencing could provide new information about these resistance-associated variants [36]. Mild gastrointestinal disorders, jaundice resulting from isolated unconjugated hyperbilirubinemia, and rash or photosensitivity were observed more frequently in the faldaprevir-treated groups than in the placebo-group. Discontinuations due to adverse events were observed in 4%–11% of the active groups. The SILEN-C1 trial showed that faldaprevir QD with pegylated interferon and ribavirin achieved consistently high SVR rates with acceptable tolerability and safety [25].

The SILEN-C2 trial was a phase 2b multicenter, randomized, double-blind study of faldaprevir in combination with pegylated interferon and ribavirin in HCV genotype 1-infected patients with nonresponse to prior pegylated interferon and ribavirin treatment (Figure 2B) [26]. In all, 290 non-cirrhotic patients were randomized into three groups. Their SVR rates, shown in Figure 2B, were 32%, 50%, and 42% in prior partial responders, and 21%, 35%, and 29% in prior null responders in the faldaprevir 240 mg QD with lead-in, 240 mg QD, and 240 mg BID with lead-in group, respectively [26]. Rates of gastrointestinal disorders, jaundice, dry skin and photosensitivity were increased at 240 mg BID compared with the 240 mg QD dose. Hyperbilirubinemia was not associated with increases in serum markers of liver injury. Faldaprevir discontinuations due to adverse events occurred in 6%, 4% and 23% of patients in the 240 mg QD with lead-in, 240 mg QD and 240 mg BID with lead-in group, respectively [26]. In previous null responders, SVR rates were 6%–36% and 25%–42% in HCV subgenotypes 1a and 1b patients, respectively. In previous partial responders, SVR rates were 21%–46% and 44%–54% in HCV subgenotypes 1a and 1b individuals, respectively. Most cases of breakthrough and relapse were owing to selection of the well-described resistance mutations R155K in HCV subgenotype 1a and D168V in HCV subgenotype 1b. The SILEN-C2 trial suggested that faldaprevir 240 mg QD with pegylated interferon and ribavirin was safe and tolerable and achieved substantial SVR rates in prior null and partial responders [26].

**Figure 3:**
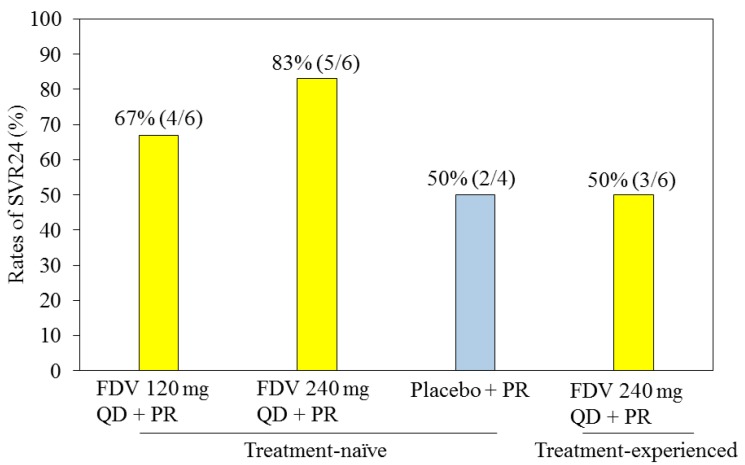
Results of a phase 2 study of faldaprevir (FDV) plus pegylated interferon (P) and ribavirin (R) in Japanese patients with chronic HCV genotype 1 infection [27]. Treatment-naive patients received FDV 120 or 240 mg QD, or placebo, plus PR for 4 weeks, then PR alone for 44 weeks. Treatment-experienced patients received FDV 240 mg QD plus PR for 4 weeks, then PR alone for 44 weeks. The rates of sustained virological response 24 weeks after therapy (SVR24) are shown.

The results from a Japanese study of faldaprevir with pegylated interferon and ribavirin [27] are shown in Figure 3. These results were similar to those reported in the treatment-naive population of SILEN-C1 [25] or those of the treatment-experienced population of SILEN-C2 [26].

### 4.2. Faldaprevir and Deleobuvir (BI 207127) with or without Ribavirin for HCV Genotype 1-Infected Patients

**Figure 4:**
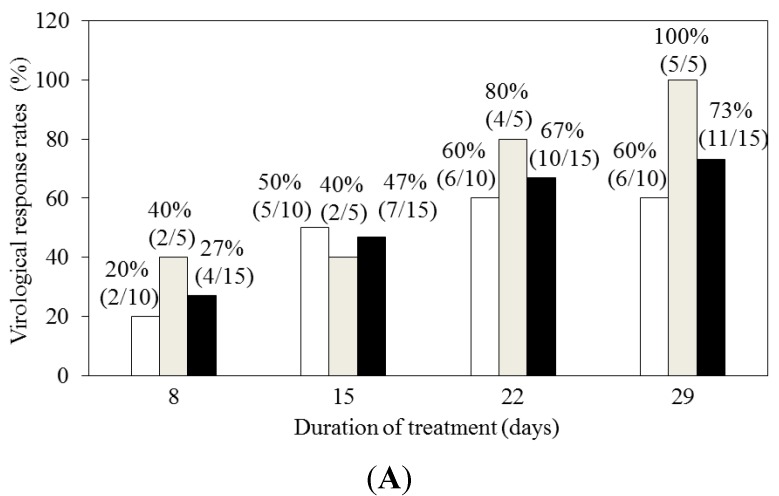

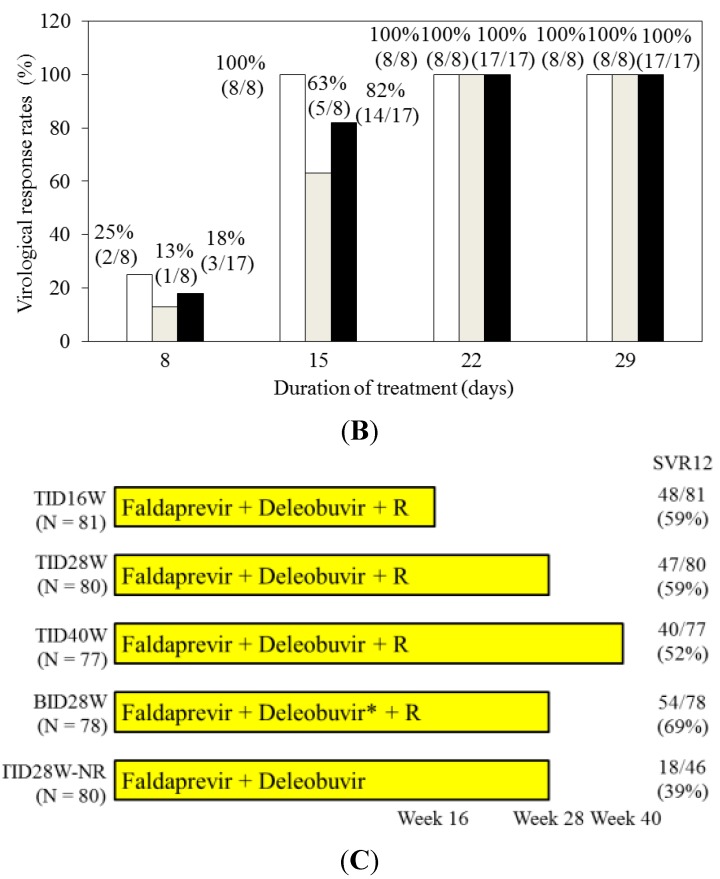
Safety and antiviral effect of Oral combinations withoUt iNnterferon in patients Diagnosed with chronic hepatitis C (SOUND) trial designs and results. (**A**,**B**) The clinical phase 1b trial (SOUND-C1) consisted of faldaprevir in combination with deleobuvir and ribavirin for 4 weeks in treatment-naive patients with chronic genotype 1 HCV [28]. (**A**) In patients treated with deleobuvir 400 mg 3 times daily + faldaprevir 120 mg once daily + ribavirin, virological response (HCV RNA level less than 25 IU/mL) by the duration of treatment and genotype. White column, HCV subgenotype 1a; gray column, HCV subgenotype 1b; black column, HCV subgenotypes 1a and 1b; (**B**) In patients treated with deleobuvir 600 mg 3 times daily + faldaprevir 120 mg once daily + ribavirin, virological response (HCV RNA level less than 25 IU/mL) by the duration of treatment and genotype; (**C**) In the clinical phase 2b trial (SOUND-C2) [29], treatment regimens were as follows: faldaprevir at a dose of 120 mg once daily and deleobuvir at a dose of 600 mg three times daily, plus ribavirin (R), for 16, 28, or 40 weeks (TID16W, TID28W, or TID40W, respectively); faldaprevir at a dose of 120 mg once daily and deleobuvir at a dose of 600 mg twice daily*, plus ribavirin (R), for 28 weeks (BID28W); and faldaprevir at a dose of 120 mg once daily and deleobuvir at a dose of 600 mg three times daily, without ribavirin, for 28 weeks (TID28W-NR). The rates of sustained virological response 12 weeks after therapy (SVR12) are shown.

Pegylated interferon and ribavirin are associated with severe adverse events, and interferon-free regimens have been desirable for the treatment of chronic HCV infection. The Safety and antiviral effect of Oral combinations withoUt iNnterferon in patients Diagnosed with chronic hepatitis C (SOUND-C1) trial was a clinical phase 1b trial that investigated the safety, antiviral effect, and pharmacokinetics of deleobuvir (a non-nucleoside polymerase inhibitor) in combination with faldaprevir (a protease inhibitor) and ribavirin for 4 weeks in treatment-naïve patients with HCV genotype 1 infection [28]. In all, 32 treatment-naïve patients were randomly assigned to groups that were given 400 or 600 mg deleobuvir 3 times daily plus 120 mg faldaprevir QD and 1000 to 1200 mg/day ribavirin for 4 weeks (Figure 4A,B). A higher rate of response was observed in HCV subgenotype 1b patients compared with HCV subgenotype 1a patients. The frequent adverse events were mild gastrointestinal disorders, rash, and photosensitivity. HCV NS3/4A protease inhibitors, HCV NS5A inhibitors, and HCV NS5B non-nucleoside polymerase inhibitors have a lower genetic barrier to resistance compared with HCV NS5B nucleoside polymerase inhibitors [37]. However, SOUND-C1 showed that the combination of faldaprevir, deleobuvir, and ribavirin has rapid and strong activity against HCV genotype 1 and did not cause serious or severe adverse events [28].

SOUND-C2 was a phase 2b, randomized, open-label trial of faldaprevir and deleobuvir with or without ribavirin for HCV genotype 1 infection [29]. A total of 362 patients were randomly assigned to 1 of 5 groups (Figure 4C). The primary end-point was a sustained virological response 12 weeks after the completion of therapy (SVR12). The rates of SVR12 were 52%–69% among patients who received interferon-free treatment with faldaprevir in combination with deleobuvir plus ribavirin. SVR12 rates did not differ significantly according to the treatment duration or dosage among the ribavirin-containing regimens. SVR12 rates were significantly higher with ribavirin-containing regimens than the ribavirin-free regimen (Figure 4C). SVR12 rates were 56%–85% in HCV subgenotype 1b *versus* 11%–47% in HCV subgenotype 1a and 58%–84% in patients with IL28B CC *versus* 33%–64% with IL28B non-CC [29]. Rash, photosensitivity, nausea, vomiting, and diarrhea were the most common adverse events. Of interest, ribavirin was necessary in these interferon-free regimens.

## 5. Conclusions

Faldaprevir in combination with pegylated interferon and ribavirin, or interferon-free treatment with faldaprevir in combination with deleobuvir plus ribavirin provides high SVR rates for HCV genotype 1 infection. The most common adverse events were gastrointestinal disorders, rash and photosensitivity. In interferon-containing regimens, most cases of breakthrough and relapse were owing to the selection of the well-described resistance mutations R155K in HCV subgenotype 1a and D168V in HCV subgenotype 1b. The interferon-free combination of faldaprevir and deleobuvir with ribavirin was effective for HCV genotype 1 infection, although further improvements will still be needed [38]. Interferon-free regimens will be useful for these patients as well as for those patients intolerant to interferon [37,39,40]. During the preparation of this manuscript, Boehringer Ingelheim has decided not to move forward in the therapeutic area of HCV.
